# The *SDF-1 rs1801157* Polymorphism is Associated with Cancer Risk: An Update Pooled Analysis and FPRP Test of 17,876 Participants

**DOI:** 10.1038/srep27466

**Published:** 2016-06-06

**Authors:** Xiang Tong, Yao Ma, Huajiang Deng, Xixi Wang, Sitong Liu, Zhipeng Yan, Shifeng Peng, Hong Fan

**Affiliations:** 1 Department of Respiratory and Critical Care Medicine, West China Hospital/West China School of Medicine, Sichuan University, Chengdu, Sichuan, 610041, China; 2 Department of Neurosurgery, The Affiliated Hospital of Luzhou Medical College, Luzhou, Sichuan, 646000, China; 3 Department of Internal Medicine, Luzhou People’s Hospital, Luzhou, Sichuan, 646000, China

## Abstract

The stromal cell derived factor-1 (*SDF-1*) *rs1801157* gene polymorphism has been implicated in susceptibility to cancer, but the results were inconclusive. The current study was to precisely investigate the association between *SDF-1 rs1801157* polymorphism and cancer risk using meta-analysis and the false positive report probability (FPRP) test. All 17,876 participants were included in the study. The meta-analysis results indicated a significant association between the *SDF-1 rs1801157* polymorphism and cancer risk. By subgroup analyses, the results detected that the *SDF-1 rs1801157* polymorphism was associated with cancer susceptibility among Asians and Caucasians. Additionally, we also found significant associations between the *SDF-1 rs1801157* polymorphism and susceptibility to different types of cancer. However, to avoid a “false positive report”, we further investigated the significant associations observed in the present meta-analysis using the FPRP test. Interestingly, the results of the FPRP test indicated that only 4 gene models were truly associated with cancer risk, especially in Asians. Moreover, we confirmed that the *SDF-1 rs1801157* gene polymorphism was only associated with lung and urologic cancer risk. In summary, this study suggested that the *SDF-1 rs1801157* polymorphism may serve as a risk factor for cancer development among Asians, especially an increased risk of urologic and lung cancers.

Cancer remains a major cause of mortality worldwide. It is estimated that the cancer death rate will be increased by 5-fold in developing countries, and the global cancer mortality is expected to increase by 104% in 2020^1^. Cancer is a multi-factorial disease that results from complex interactions between numerous genetic and environmental factors^2^,^3^. In recent years, an increasing number of studies has focused on the association between chemokine gene variants and malignancy susceptibility. Among them, the stromal cell derived factor-1 (*SDF-1*) gene, also known as CXC chemokine ligand 12 (CXCL12), has been widely studied^4^,^5^,^6^. The *SDF-1* gene is located on chromosome 10q11.1 and spans 10 kb^7^. The *SDF-1* gene mainly encodes a CXC angiogenic chemokine^8^. One important *SDF-1* gene polymorphism named *rs1801157* (G801A) involves a guanine to adenine (G→C) substitution at base pair 801 of the 3′-untranslated region of *SDF-1* gene^9^.

Previous studies have indicated that *SDF-1* participates in several biological activities, including neuronal and cardiogenesis development, haematopoiesis and lymphocyte trafficking^10^. Furthermore, growing evidence suggests that the *SDF-1 rs1801157* polymorphism plays an important role in the pathogenesis of cancer. Razmkhah *et al*. reported that the *SDF-1 rs1801157* polymorphism increased the risk of breast and lung cancer^11^,^12^. Kucukgergin and co-workers showed that the *SDF-1 rs1801157* polymorphism was associated with bladder cancer susceptibility^13^. Hirata *et al*. reported that the *SDF-1 rs1801157* polymorphism is potentially associated with an increased risk of prostate cancer among Japanese individuals^14^. However, Petersen *et al*. did not identify an association between the *SDF-1 rs1801157* polymorphism and prostate cancer susceptibility^15^, and similar negative results were also detected by Tee and colleagues^16^.

The results of those genetic association studies were inconclusive. Moreover, a single study may be insufficient to detect a small effect of the *SDF-1 rs1801157* gene polymorphism on cancer susceptibility, especially when the sample size is relatively small. Considering the crucial role of the *SDF-1 rs1801157* gene polymorphism in the pathogenesis of cancer, we performed a meta-analysis to accurately investigate the association of the *SDF-1 rs1801157* gene polymorphism with cancer risk. Furthermore, to avoid a “false positive report”, we further assessed the significant associations observed in the present meta-analysis using the false positive report probability (FPRP) test. To our knowledge, this study is most recent and accurate meta-analysis exploring the *SDF-1 rs1801157* gene polymorphism in cancer susceptibility.

## Results

### Study characteristics

**Figure 1:**
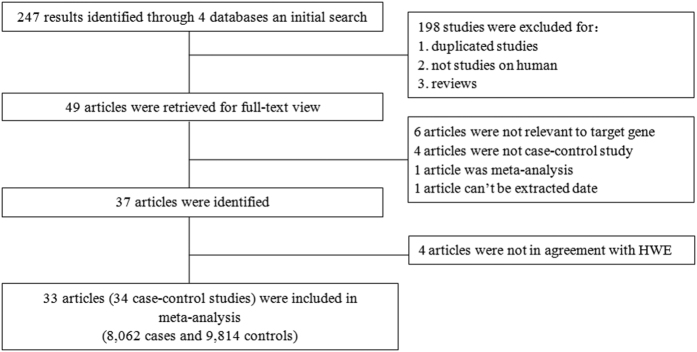


As indicated in the flow diagram (Fig. 1), 247 articles were identified after an initial search. After reading the titles and abstracts, we excluded 198 articles. The remaining 49 articles were further screened by a full-text review. Six articles were excluded because they investigated other *SDF-1* gene polymorphisms (e.g., rs2839693 and rs1065297) rather than the *rs1801157* polymorphism. Four articles were excluded because they were not case-control studies. One article was not included because it was a meta-analysis, and one article was excluded because the available data could not be extracted to further assess the effect size. Therefore, all 37 articles were identified^4^,^5^,^6^,^11^,^12^,^13^,^14^,^15^,^16^,^17^,^18^,^19^,^20^,^21^,^22^,^23^,^24^,^25^,^26^,^27^,^28^,^29^,^30^,^31^,^32^,^33^,^34^,^35^,^36^,^37^,^38^,^39^,^40^,^41^,^42^,^43^,^44^. However, according to the results of the HWE test, 4 articles^18^,^38^,^39^,^44^ were excluded because they did not achieve HWE in the control group. Finally, a total of 34 eligible case-control studies from 33 articles^4^,^5^,^6^,^11^,^12^,^13^,^14^,^15^,^16^,^17^,^19^,^20^,^21^,^22^,^23^,^24^,^25^,^26^,^27^,^28^,^29^,^30^,^31^,^32^,^33^,^34^,^35^,^36^,^37^,^40^,^41^,^42^,^43^ were included in the study. Among them, 18 articles were performed on Caucasians^4^,^6^,^15^,^17^,^19^,^20^,^21^,^22^,^23^,^24^,^27^,^28^,^29^,^31^,^33^,^34^,^40^,^43^, 13 articles on Asians^5^,^11^,^12^,^13^,^14^,^16^,^25^,^26^,^30^,^32^,^35^,^36^,^37^, and two studies on individuals of mixed ethnicity^41^,^42^. In addition, based on the quality score, eleven studies were considered low quality. The characteristics of included studies are summarized in Tables 1 and 2.

### Meta-analysis results

**Figure 2:**
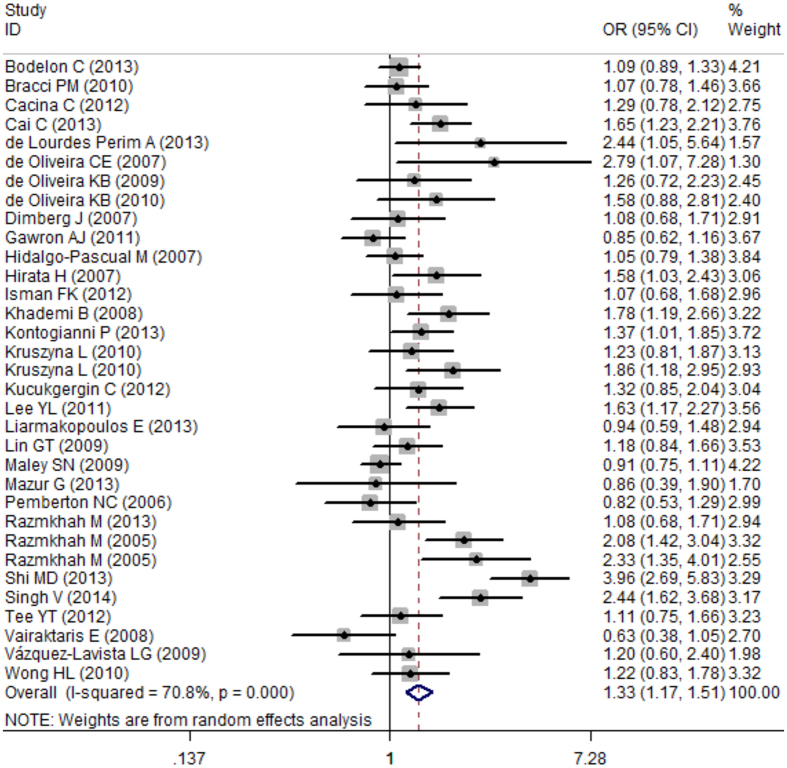


**Figure 3:**
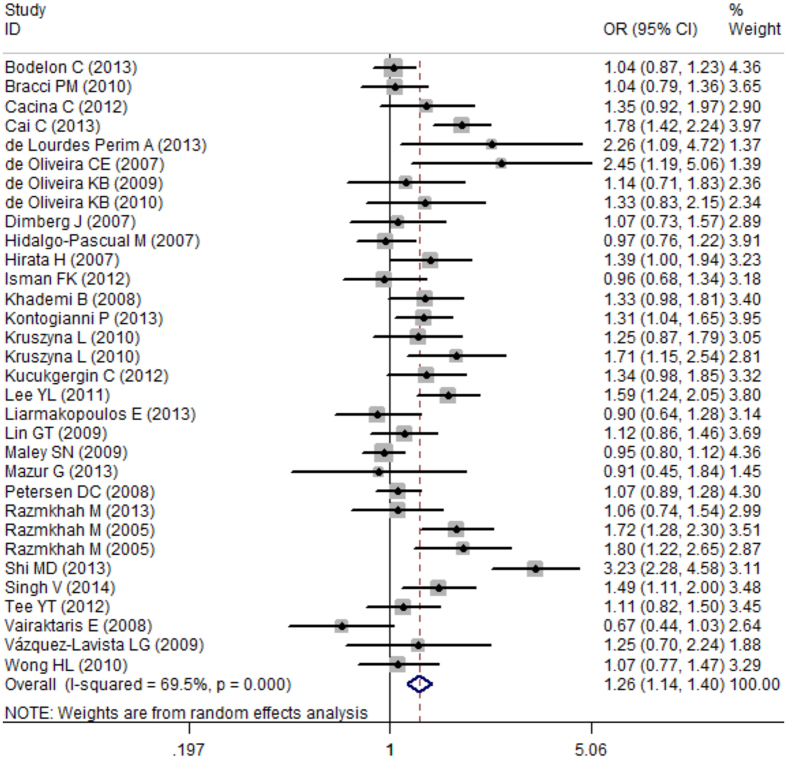


In total, 17,876 participants (8,062 cases and 9,814 controls) from 34 case-control studies were included in the current meta-analysis assessing the relationship between the *SDF-1 rs1801157* polymorphism and cancer risk. In the overall meta-analysis, the results suggested a significant association between the *SDF-1 rs1801157* polymorphism and cancer susceptibility in the recessive model (AA vs. AG + GG, OR: 1.28, 95% CI: 1.11–1.47, P = 0.001), a co-dominant model (AA vs. GG, OR: 1.43, 95% CI: 1.24–1.65, P < 0.001) as assessed by a fixed-effect model (I^2^ = 47%, 49.7%, respectively) and other models (AA + AG vs. GG, OR: 1.33, 95% CI: 1.17–1.51, P < 0.001; AG vs. GG, OR: 1.35, 95% CI: 1.19–1.54, P < 0.001; A vs. G, OR: 1.26, 95% CI: 1.14–1.40, P < 0.001) (Figs 2 and 3) as assessed by a random-effect model (I^2^ = 70.8%, 66.4%, 69.5%, respectively) (Table 3).

In the subgroup analyses of ethnicity, the meta-analysis results indicated a strong association between the *SDF-1 rs1801157* polymorphism and cancer susceptibility among Asians (AA + AG vs. GG, OR: 1.63, 95% CI: 1.34–1.98, P < 0.001) and a weak association in Caucasians (AG vs. GG, OR: 1.10, 95% CI: 1.00–1.21, P = 0.05), but no association in mixed populations using different genetic models (Table 3). Additionally, we also conducted subgroup analyses of cancer types. As shown in Table 3, significant associations between the *SDF-1 rs1801157* polymorphism and breast cancer, urologic cancer, hematologic malignancy and lung cancer susceptibility were found in different genetic models. However, no association was observed between the *SDF-1 rs1801157* polymorphism and risk of head and neck cancer, gynaecological cancer and digestive system cancer (Table 3).

### Publication bias and sensitivity analysis

**Figure 4:**
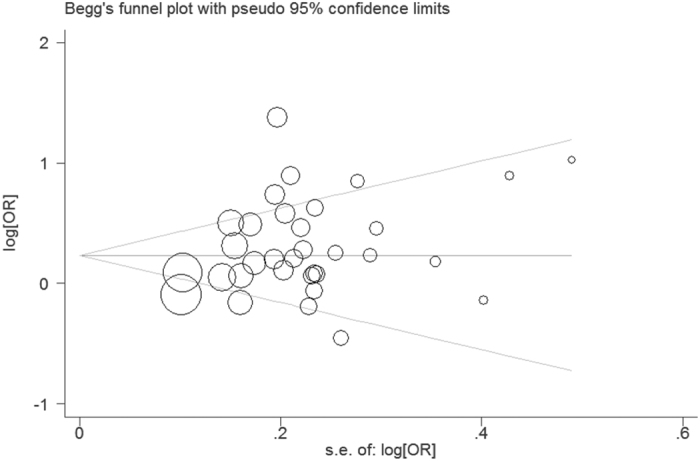


**Figure 5:**
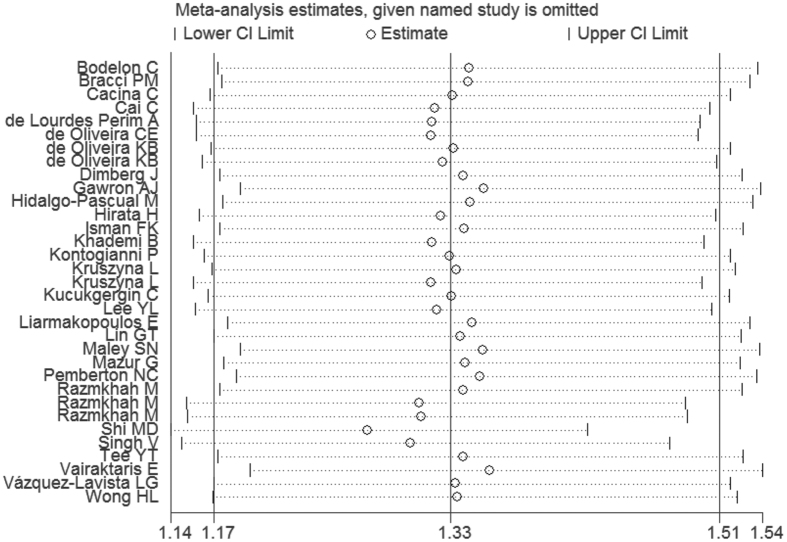


The funnel plot is a symmetrical inverted funnel (Fig. 4). No publication biases were noted using Begg’s (P = 0.329) and Egger’s tests (P = 0.082). Additionally, to further investigate the possible source of heterogeneity, we executed a sensitivity analysis by sequentially excluding studies from the meta-analysis to investigate the influence of each study on the pooled results. The sensitivity analysis results reported that the pooled ORs were not materially altered, suggesting the stability of our meta-analysis (Fig. 5).

### FPRP test results

Moreover, we further investigated the significant associations (P < 0.05) observed in the present meta-analysis using the FPRP test. As listed in Table 4, the FPRP test results revealed that all 4 gene models (AA + AG vs. GG, AA vs. GG, AG vs. GG, A vs. G) of the *SDF-1 rs1801157* gene polymorphism were truly associated with cancer risk (FPRP = 0.011, 0.001, 0.008, 0.017, respectively) at an *a priori* probability level of 0.001 with an OR of 1.5, especially in Asians. In addition, according to the results of the FPRP test, we confirmed that the *SDF-1 rs1801157* gene polymorphism was only associated with lung cancer risk in the allele model (A vs. G, FPRP = 0.019), whereas the polymorphism was associated with susceptibility to urologic cancer in the dominant gene model (AA + AG vs. GG, FPRP = 0.001) at an *a priori* probability level of 0.001 and an OR of 1.5.

## Discussion

Cancer has high morbidity and mortality worldwide. Previous studies have suggested that cancer risk factors include an unhealthy life style, environmental pollution, radiation, infection, and immunity dysfunction^45^,^46^,^47^. Additionally, studies have increasingly focused on genetic factors^48^,^49^,^50^. In recent years, numerous studies reported an association between the *SDF-1 rs1801157* polymorphism and cancer susceptibility, but the results were inconsistent or inconclusive. In addition, a single study may lack sufficient power to detect the potentially small effect of the *SDF-1 rs1801157* polymorphism on cancer, especially when the sample size is relatively small. Meta-analysis is a useful method for investigating cancer associations with genetic factors because this method uses a quantitative approach by combining the results of different studies on the same topic, potentially providing more reliable conclusions. Thus, we conducted a pooled analysis to investigate the association of the *SDF-1 rs1801157* polymorphism and risk of cancer.

In fact, two recently published meta-analyses^51^,^52^ investigated the association between the *SDF-1 rs1801157* gene polymorphism and cancer risk. One study^51^ included 29 papers involving 4932 cases and 7917 controls, and another article^52^ only included 4,435 cancer cases and 6,898 controls from 25 studies. Additionally, the two meta-analyses did not assess the quality of the included studies. Furthermore, the previous two studies did not use the FPRP test to explore truly significant associations, and the results of those studies were potentially unable to reflect the true association between the *SDF-1 rs1801157* gene polymorphism and cancer risk. Therefore, we performed a meta-analysis and FPRP test to assess the association between the *SDF-1 rs1801157* gene polymorphism and cancer risk.

In total, 8,062 cases and 9,814 controls were included in the current meta-analysis. Based on our overall meta-analysis and FPRP tests, we found that the *SDF-1 rs1801157* polymorphism obviously increased the risk of cancer. Additionally, we identified significant heterogeneity between studies in the present meta-analysis. Although heterogeneity can be considered using the random-effect model, heterogeneity increases the probability of type-I errors. The following factors may have contributed to the significant heterogeneity: 1) different demographic and genetic characteristics of Caucasian, Asian and mixed populations; 2) different types of cancer may be caused by different mechanisms; 3) different stages of cancer in each study among the studied cancer patients; 4) different genotyping methods of each study; and 5) different qualities among included studies.

Hence, to identify the cause of heterogeneity, we conducted subgroup analyses based on ethnicity and cancer type. The results suggested that the *SDF-1 rs1801157* gene polymorphism strongly increased the cancer risk among Asians, whereas the heterozygote genotype (AG) of the *SDF-1 rs1801157* gene polymorphism only weakly increased cancer susceptibility in Caucasians. Unfortunately, the *SDF-1 rs1801157* gene polymorphism did not increase or decrease the cancer risk among mixed populations. Furthermore, the results of sub-group analyses after stratification based on cancer type indicated that the *SDF-1 rs1801157* polymorphism increased the risk of breast cancer, urologic cancer, lung cancer, head and neck cancer, and haematological malignancies. However, the *SDF-1 rs1801157* polymorphism did not influence gynaecological cancer and digestive system cancer susceptibility. Despite the fact that the heterogeneity was still detected when we performed stratified analyses based on ethnicity and cancer type, the subgroup analyses may provide more precise results compared with the overall analysis.

Several previous studies indicated that the published “statistically significant” results for genetic variants were false-positive findings, even in large and well-designed studies^53^,^54^. Fortunately, we could use the FPRP test to estimate true significant associations in the current meta-analysis. The FPRP is calculated from the statistical power of the test, the observed p-value, and a given *a priori* probability for the association.

Therefore, based on the positive results of the current meta-analysis, we further investigated whether a significant association between the *SDF-1 rs1801157* gene polymorphism and cancer risk is “noteworthy”. Interestingly, the FPRP test results revealed that the *SDF-1 rs1801157* gene polymorphism actually increased cancer susceptibility. Additionally, the FPRP test also demonstrated that the *SDF-1 rs1801157* gene polymorphism could increase cancer risk among Asians, but it did not increase cancer risk in Caucasians. Moreover, the results of the FPRP test confirmed that the *SDF-1 rs1801157* gene polymorphism increased the risk of lung cancer and urologic cancer. Surprisingly, the significant associations with breast cancer, head and neck cancer, and haematological malignancies in the present meta-analysis were false positive at the a priori probability level of 0.001 and an OR of 1.5. Some of the discoveries may be false-positive findings due to the limited sample size in each stratum, so it is important to perform FPRP analysis to avoid these findings, especially when the sample size is not of sufficient size.

The mechanism by which the *SDF-1 rs1801157* polymorphism affects cancer risk is unclear. Previous studies indicated that *SDF-1* is a chemokine that plays a pivotal role in different stages of cancer development and metastasis^55^,^56^. Combined with previous results^57^ and our present meta-analysis results, we put forward a simple hypothesis that the *SDF-1 rs1801157* polymorphism, which has a G > A transition in the 3′-UTR, may have an important regulatory function of up-regulating *SDF-1* production and the high serum concentrations of *SDF-1* might contribute to cancer susceptibility. Finally, people who carried the variant A allele are more likely to develop cancer, especially urologic cancer and lung cancer.

Several limitations in the present meta-analysis should be noted. First, the published studies include small sample sizes, and only published articles were included in a few databases. Thus, these limitations might lead to additional biases or overall overestimated associations. Moreover, a language bias potentially also occurred because the including articles were only published in English and Chinese. Second, given the lack sufficient data for each included study, we failed to perform further subgroup analysis to investigate the risk factors of cancer, such as gender, gene-environment/gene-gene interactions, life style and age. Third, a small number of studies were included in the subgroup analysis to investigate the association between the *SDF-1 rs1801157* polymorphism and head and neck cancer risk, so we must be cautious when referring to the pooled results. A study with larger samples is needed to confirm the results in the future. Despite these limitations, we minimized the likelihood of bias throughout the entire process by creating a detailed protocol and carefully performing study identification, statistical analysis and data selection. Thus, the reliability of the results is guaranteed.

In conclusion, the current study suggested that the *SDF-1 rs1801157* polymorphism may contribute to the risk of cancer in Asians. In particular, the polymorphism could increase urologic and lung cancer susceptibility. More well-designed studies with larger sample sizes focusing on ethnicities or cancer types should be conducted to confirm the results in the future.

## Methods

### Study selection

A systematic literature search of the PubMed, Embase, and Wanfang databases and the China National Knowledge Internet (CNKI) was performed to identify studies involving the relationship between the *SDF-1 rs1801157* gene polymorphism and cancer susceptibility, with the last updated search being conducted on May 14, 2015. The following key words were used: (stromal cell derived factor-1 OR *SDF-1* OR CXC chemokine ligand 12 OR CXCL12) AND (cancer OR tumour OR neoplasm OR malignancy OR leukaemia OR myeloma OR sarcoma OR lymphoma) AND (polymorphism OR mutation OR variant). The language was restricted to English and Chinese.

### Inclusion and exclusive criteria

The inclusion criteria were defined as follows: 1) the study should be a case-control study; 2) the association between the *SDF-1 rs1801157* polymorphism and cancer risk should be evaluated; 3) the available genotype or allele frequencies for counting the odds ratio (OR) and 95% confidence interval (CI) should be provided in the study; 4) the study should evaluate humans; and 5) the genotype distributions of control cohorts should be in with Hardy-Weinberg equilibrium (HWE). The following exclusive criteria were adopted: 1) the study was not designed as a case-control study; 2) review, abstract or overlapping study; and 3) the study cannot provide available genotype or allele frequencies for determining the effect size.

### Quality score evaluation

The qualities of included studies were assessed by the Newcastle-Ottawa Scale (case control study). The scale estimates quality based on three aspects: selection, comparability and exposure in the study. The total score ranged from 0 to 9, and a score greater than 6 was considered to represent high quality. In addition, we assessed the quality of the studies in a consensus meeting with all authors.

### Data extraction

The independent authors (Xiang Tong and Huajiang Deng) collected detailed data from each study according to the inclusive criteria. If a disagreement was encountered, the third author (Xixi Wang) evaluated the articles. For each study, the first author, publication year, country, ethnicity, number of case and control groups, type of cancer, genotype and allele distribution, and genotyping method were extracted. The information is listed in Tables 1 and 2.

### Statistical methods

The OR and 95% CI were used to estimate the effect strength of association between the *SDF-1 rs1801157* polymorphism and cancer susceptibility. We calculated heterogeneity using the χ^2^-based Q-test and I-squared (I^2^) statistics tests. The pooled OR was assessed by the random-effect model when the heterogeneity was considered to be statistically significant (I^2^ > 50% and P  < 0.10); alternatively, the fixed-effect model was applied. We explored the association between the *SDF-1 rs1801157* polymorphism and cancer risk in different gene models (AA + AG vs. GG, AA vs. AG + GG, AA vs. GG, AG vs. GG and A vs. G). To evaluate the effects of ethnicity and type of cancer, we also performed subgroup analyses based on ethnicity group and type of cancer.

In addition, to evaluate whether significant associations (P < 0.05) detected in the present study are “noteworthy”, we further calculated the FPRP value at a probability level of 0.001 and an OR of 1.5 58. As suggested by the previous study^59^, we set a FPRP cut-off value of 0.2, and only FPRP results < 0.2 were considered to be “noteworthy”. Publication bias was assessed by several methods. A visual inspection of asymmetry in funnel plots was performed. Furthermore, the Begger’s and Egger’s tests were used to assess the publication bias^60^. Additionally, HWE was assessed in each study using the Chi-square before the present meta-analysis was performed. All analyses were conducted using STATA 11.0 software.

## Additional Information

**How to cite this article**: Tong, X. *et al*. The *SDF-1 rs1801157* Polymorphism is Associated with Cancer Risk: An Update Pooled Analysis and FPRP Test of 17,876 Participants. *Sci. Rep.* **6**, 27466; doi: 10.1038/srep27466 (2016).

## Figures and Tables

**Table 1:** Characteristics of case-control studies included in the meta-analysis.

First author	Year	Country	Ethnicity	Type	Sample size	Genotyping method	HWE	Score
					Case/Control			
Bodelon C	2013	USA	Caucasian	BC	840/801	IGGMP	Yes	6
Bracci PM	2010	USA + UK	Caucasian	NHL	255/511	IGGMP	Yes	8
Cacina C	2012	Turkey	Asian	EC	113/139	PCR-RFLP	Yes	6
Cai C	2013	China	Asian	RC	322/402	PCR-RFLP	Yes	7
de Lourdes Perim A	2013	Brazil	Caucasian	ALL	54/58	PCR-RFLP	Yes	6
de Oliveira CE	2007	Brazil	Caucasian	CML	25/60	PCR-RFLP	Yes	5
de Oliveira KB	2009	Brazil	Caucasian	BC	103/97	PCR-RFLP	Yes	5
de Oliveira KB	2010	Brazil	Caucasian	NHL + HL	106/90	PCR-RFLP	Yes	5
Dimberg J	2007	Sweden	Caucasian	CRC	151/141	PCR-RFLP	Yes	5
Gawron AJ	2011	Poland	Caucasian	GC	292/414	TaqMan	Yes	7
Hidalgo-Pascual M	2007	Spain	Caucasian	CRC	349/516	Real-time PCR	Yes	6
Hirata H	2007	Japan	Asian	PC	167/167	PCR-RFLP	Yes	5
Isman FK	2012	Turkey	Asian	PC	152/149	PCR-RFLP	Yes	7
Khademi B	2008	Iran	Asian	HC + NC	156/262	PCR-RFLP	Yes	6
Kontogianni P	2013	Greece	Caucasian	BC	261/480	PCR-RFLP	Yes	6
Kruszyna L	2010	Poland	Caucasian	BC	193/199	PCR-RFLP	Yes	6
Kruszyna L	2010	Poland	Caucasian	LAC	118/250	PCR-RFLP	Yes	6
Kucukgergin C	2012	Turkey	Asian	BLC	142/197	PCR-RFLP	Yes	7
Lee YL	2011	China	Asian	LC	247/328	PCR-RFLP	Yes	7
Liarmakopoulos E	2013	Greece	Caucasian	GC	88/480	PCR-RFLP	Yes	6
Lin GT	2009	China	Asian	BC	220/334	PCR-RFLP	Yes	4
Maley SN	2009	USA	Caucasian	CC	899/820	TaqMan	Yes	7
Mazur G	2013	Poland	Caucasian	MM	54/75	PCR-RFLP	Yes	4
Pemberton NC	2006	UK	Caucasian	CLL	323/108	PCR-RFLP	Yes	5
Petersen DC	2008	Australia	Caucasian	PC	815/727	TaqMan	Yes	6
Razmkhah M	2013	Iran	Asian	GC + CC	233/262	PCR-RFLP	Yes	4
Razmkhah M	2005	Iran	Asian	BC	278/181	PCR-RFLP	Yes	6
Razmkhah M	2005	Iran	Asian	LC	72/262	PCR-RFLP	Yes	4
Shi MD	2013	China	Asian	CRC	258/300	DHPLC	Yes	5
Singh V	2014	India	Asian	BLC	200/200	PCR-RFLP	Yes	7
Tee YT	2012	China	Asian	CC	137/337	PCR-RFLP	Yes	7
Vairaktaris E	2008	Europe	Caucasian	OC	159/101	PCR-RFLP	Yes	6
Vázquez-Lavista LG	2009	Mexico	Mixed	BLC	47/126	PCR-RFLP	Yes	7
Wong HL	2010	USA	Mixed	NHL	233/240	TaqMan	Yes	7

BC = breast cancer; EC = endometrial cancer; RC = renal cancer; ALL = acute lymphatic leukaemia; CML = chronic myeloid leukaemia; NHL non-Hodgkin’s lymphoma; HL = Hodgkin’s lymphoma; CRC = colorectal cancer; GC = gastric cancer; PC = prostate cancer; HC = head cancer; NC = neck cancer; LAC = laryngeal cancer; BLC = bladder cancer; LC = lung cancer; CC = cervical cancer; MM = multiple myeloma; CLL = chronic lymphocytic leukaemia; OC = ovarian cancer; IGGMP = Illumina Golden Gate multiplex platform; PCR-RFLP = polymerase chain reaction-restricted fragment length polymorphism; DHPLC  = denaturing high performance liquid chromatography.

**Table 2:** Distributions of the *SDF-1 rs1801157* allele and genotypes in cases and controls.

First author	Year	Case					Control				
		AA	AG	GG	A	G	AA	AG	GG	A	G
Bodelon C	2013	29	288	523	346	1334	35	251	515	321	1281
Bracci PM	2010	6	85	164	97	413	14	161	336	189	833
Cacina C	2012	12	52	49	76	150	6	64	69	76	202
Cai C	2013	61	111	150	233	411	29	136	237	194	610
de Lourdes Perim A	2013	3	18	33	24	84	1	11	46	13	103
de Oliveira CE	2007	4	11	10	19	31	3	18	39	24	96
de Oliveira KB	2009	3	41	59	47	159	4	32	61	40	154
de Oliveira KB	2010	5	43	58	53	159	5	26	59	36	144
Dimberg J	2007	5	62	84	72	230	4	56	81	64	218
Gawron AJ	2011	99		193	NA	NA	156		258	NA	NA
Hidalgo-Pascual M	2007	9	128	212	146	552	25	172	319	222	810
Hirata H	2007	17	78	72	112	222	13	63	91	89	245
Isman FK	2012	17	66	69	100	204	22	57	70	101	197
Khademi B	2008	8	84	64	100	212	20	97	145	137	387
Kontogianni P	2013	29	118	114	176	346	35	198	247	268	692
Kruszyna L	2010	9	61	123	79	307	5	58	136	68	330
Kruszyna L	2010	3	46	69	52	184	2	67	181	71	429
Kucukgergin C	2012	26	58	58	110	174	23	80	94	126	268
Lee YL	2011	36	112	99	184	310	21	136	171	178	478
Liarmakopoulos E	2013	6	43	39	55	121	46	229	205	321	639
Lin GT	2009	16	98	106	130	310	23	136	175	182	486
Maley SN	2009	30	276	593	336	1462	23	274	523	320	1320
Mazur G	2013	1	13	39	15	91	1	21	53	23	127
Pemberton NC	2006	114		209	NA	NA	43		65	NA	NA
Petersen DC	2008	NA	NA	NA	326	1304	NA	NA	NA	276	1178
Razmkhah M	2013	18	87	128	123	343	8	39	62	55	163
Razmkhah M	2005	34	139	105	207	349	13	67	101	93	269
Razmkhah M	2005	9	38	25	56	88	20	97	145	137	387
Shi MD	2013	4	113	141	121	395	0	52	248	52	548
Singh V	2014	9	132	59	150	250	16	83	101	115	285
Tee YT	2012	16	58	63	90	184	33	140	164	206	468
Vairaktaris E	2008	4	51	104	59	259	5	41	55	51	151
Vázquez-Lavista LG	2009	3	15	29	21	73	4	39	83	47	205
Wong HL	2010	7	78	148	92	374	13	64	163	90	390

NA = not available.

**Table 3:** Summary the results of the total and subgroup analyses in different genetic models.

Variables	AA + AG vs. GG			AA vs. AG + GG			AA vs. GG			AG vs. GG			A vs. G		
	**OR**	**95% CI**	**P**	**OR**	**95% CI**	**P**	**OR**	**95% CI**	**P**	**OR**	**95% CI**	**P**	**OR**	**95% CI**	**P**
Total	1.33	1.17–1.51	<0.001	1.28	1.11–1.47	0.001	1.43	1.24–1.65	<0.001	1.35	1.19–1.54	<0.001	1.26	1.14–1.40	<0.001
Ethnicities															
Caucasian	1.07	0.98–1.16	0.15	1.03	0.82–1.29	0.81	1.07	0.85–1.35	0.55	1.10	1.00–1.21	0.05	1.07	1.00–1.15	0.06
Asian	1.63	1.34–1.98	<0.001	1.42	1.05–1.93	0.02	1.71	1.28–2.30	<0.001	1.58	1.28–1.95	<0.001	1.45	1.25–1.68	<0.001
Mixed	1.21	0.87–1.69	0.26	0.92	0.25–3.34	0.90	0.82	0.37–1.81	0.62	1.28	0.90–1.82	0.16	1.11	0.83–1.47	0.48
Types															
Breast cancer	1.26	1.11–1.44	<0.001	1.21	0.93–1.59	0.16	1.36	1.03–1.79	0.03	1.26	1.10–1.44	<0.001	1.20	1.08–1.33	<0.001
Gynaecological cancer	0.98	0.83–1.16	0.81	1.36	0.93–1.99	0.12	1.36	0.92–2.01	0.13	0.94	0.79–1.12	0.52	1.03	0.89–1.18	0.71
Urologic cancer	1.56	1.32–1.85	<0.001	1.34	0.75–2.38	0.32	1.62	0.97–2.69	0.06	1.46	1.10–1.93	<0.001	1.31	1.09–1.57	<0.001
Digestive system cancer	1.25	0.79–1.98	0.34	0.83	0.55–1.27	0.40	0.88	0.57–1.35	0.55	1.38	0.82–2.33	0.23	1.26	0.80–1.97	0.32
Hematologic malignancy	1.17	0.97–1.41	0.09	0.95	0.57–1.59	0.98	1.06	0.63–1.78	0.83	1.29	1.04–1.59	0.02	1.18	0.99–1.40	0.07
Lung cancer	1.80	1.36–2.39	<0.001	2.24	1.41–3.57	<0.001	2.86	1.75–4.69	<0.001	1.62	1.20–2.18	<0.001	1.65	1.34–2.04	<0.001
Head and neck cancer	1.3	0.68–2.48	0.43	0.76	0.40–1.46	0.41	0.92	0.47–1.80	0.81	1.35	0.70–1.61	0.37	1.16	0.71–1.91	0.55

OR = odds ratio; CI = confidence interval.

**Table 4:** The results of the FPRP tests in each gene models.

Gene models	OR (95% CI)	Power	Prior Probability = 0.001
AA + AG vs. GG		OR = 1.50	FPRP
Total	1.33 (1.17–1.51)	0.968	0.011
Asians	1.63 (1.34–1.98)	0.201	0.004
Breast cancer	1.26 (1.11–1.44)	0.995	0.41
Urologic cancer	1.56 (1.32–1.85)	0.326	0.001
Lung cancer	1.80 (1.36–2.39)	0.104	0.318
AA vs. AG + GG			
Total	1.28 (1.11–1.47)	0.988	0.323
Asian	1.42 (1.05–1.93)	0.637	0.975
Lung cancer	2.24 (1.41–3.57)	0.046	0.938
AA vs. GG			
Total	1.43 (1.24–1.65)	0.744	0.001
Asians	1.71 (1.28–2.30)	0.193	0.668
Breast cancer	1.36 (1.03–1.79)	0.758	0.974
Lung cancer	2.86 (1.75–4.69)	0.005	0.856
AG vs. GG			
Total	1.35 (1.19–1.54)	0.942	0.008
Caucasians	1.10 (1.00–1.21)	1	0.980
Asians	1.58 (1.28–1.95)	0.314	0.061
Breast cancer	1.26 (1.10–1.44)	0.995	0.410
Urologic cancer	1.46 (1.10–1.93)	0.575	0.932
Hematologic malignancy	1.29 (1.04–1.59)	0.921	0.949
Lung cancer	1.62 (1.20–2.18)	0.306	0.826
A vs. G			
Total	1.26 (1.14–1.40)	0.999	0.017
Asians	1.45 (1.25–1.68)	0.674	0.001
Breast cancer	1.20 (1.08–1.33)	1	0.339
Urologic cancer	1.31 (1.09–1.57)	0.929	0.788
Lung cancer	1.65 (1.34–2.04)	0.189	0.019

OR = odds ratio; FPRP = false positive report probability.
